# Enhancing emergency endoscopy efficiency with an additional suction channel: In vitro assessment

**DOI:** 10.1055/a-2752-2380

**Published:** 2025-12-09

**Authors:** André Sasse, Thomas Roland Heiduk, Marvin Scheunemann, Lukas Hiebel, Richard F Knoop, Marius Adler, Ali Seif Amir Hosseini, Edris Wedi, Imke Amanzada, Volker Ellenrieder, Golo Petzold, Ahmad Amanzada

**Affiliations:** 1 27177 Clinic for Gastroenterology, Gastrointestinal Oncology and Endocrinology, University Medical Center Göttingen, Göttingen, Germany; 2 High Precision Components Witten GmbH, Witten, Germany; 3 Avelios Medical GmbH, Munich, Germany; 4 27177 Department of Clinical and Interventional Radiology, University Medical Center Göttingen, Göttingen, Germany; 5 Division of Gastroenterology, Gastrointestinal Oncology and Interventional Endoscopy, Sana Clinic Offenbach, Offenbach, Germany; 6 27177 Department of Psychiatry and Psychotherapy, University Medical Center Göttingen, Göttingen, Germany

**Keywords:** Endoscopy Upper GI Tract, Non-variceal bleeding, Portal hypertension and variceal bleeding, Quality and logistical aspects, Performance and complications

## Abstract

**Background and study aims:**

Emergency endoscopic interventions for upper gastrointestinal hemorrhage are frequently hampered by presence of blood clots and food debris. This study aimed to assess whether integration of an additional suction channel (ASC) onto a standard gastroscope enhances efficiency of aspirating clots and viscous fluids.

**Patients and methods:**

A 5.3-mm suction catheter was used as an ASC mounted on a 2.8-mm standard gastroscope. Suction efficacy was evaluated using gastroscopes with working channel diameters of 2.8 mm, 3.7 mm, and 6 mm in vitro. Defined volumes of water, fruit yogurt, and coagulated blood were aspirated, and time required for complete evacuation was measured. Each setup was tested with and without the BioVac system.

**Results:**

The ASC significantly enhanced suction performance across all test media. Notably, the 2.8-mm gastroscope with ASC outperformed all other configurations in aspirating water and yogurt. For clotted blood, the ASC significantly improved evacuation times compared with all other setups besides 6-mm + BioVac.

**Conclusions:**

A standard gastroscope equipped with an ASC significantly enhances suction performance in an in vitro model, outperforming gastroscopes with larger working channels. These findings warrant further validation in an ex vivo model to determine their clinical applicability.

## Introduction

Gastrointestinal bleeding is a common and potentially life-threatening condition, representing a major cause of morbidity and mortality globally. Prompt identification of the bleeding source and quick therapeutic intervention are critical for effective patient management. Endoscopic techniques, including esophagogastroduodenoscopy (EGD) and colonoscopy, remain the cornerstone of both diagnostic and therapeutic approaches for gastrointestinal bleeding. Despite significant advances in endoscopic technology, several challenges persist, particularly the efficient removal of blood, clots, and other debris to establish and maintain a clear visual field of potential bleeding sites during procedures. These obstacles can delay diagnostic and compromise potential treatment outcomes, especially in high-risk patients with severe or ongoing hemorrhage 1 2 .

Over the years, different innovations have sought to address this issue. Further research investigated specialized endoscopes with larger working channels compared to standard gastroscopes 3 4 , oral or nasogastric lavage 5 6 7 , suction hose systems with integrated pumps 8 9 , and administration of prokinetics prior to emergency endoscopy 10 11 12 . Furthermore, several case reports have documented use of polypectomy snares, grasping forceps, ethanol injection balloon catheters, and overtubes for clot removal 13 14 15 16 17 18 19 20 . Integration of additional suction channels into endoscopic systems is among these developments, designed to enhance fluid evacuation and optimize visualization during gastrointestinal endoscopic procedures. The idea behind additional suction channels is based on the hypothesis that improving efficiency of fluid evacuation could facilitate rapid and effective identification of bleeding sources, thereby potentially reducing procedure duration and improving clinical outcome 21 . However, despite the theoretical advantages, there is a lack of data evaluating the performance and practical implications of such modifications, particularly in the context of emerging endoscopic platforms designed for management of gastrointestinal bleeding 17 .

This study investigated the effect of an additional suction channel (ASC) incorporated into a novel endoscopic system using an in vitro model simulating gastrointestinal bleeding. It assessed the impact of this modification on suction efficiency, fluid clearance, and ability to maintain clear visualization in the presence of simulated gastrointestinal bleeding. Given the clinical significance of rapid and accurate diagnosis in gastrointestinal bleeding, understanding functional improvements provided by this modification may offer valuable insights for optimizing endoscopic procedures and ultimately improving patient outcome in this critical domain.

## Methods

### Study design

This study was a prospective in vitro trial conducted at the Department of Gastroenterology, Gastrointestinal Oncology and Endocrinology of the University Medical Center Göttingen in Germany. Institutional Review Board (IRB) approval was not required. Blood collection for research purposes was approved by the IRB (8/7/20). All volunteers provided informed consent in accordance with the Declaration of Helsinki and the approval of the IRB.

### Materials and methods

Endoscope setup and suction process.Video 1

**Fig. 1:**
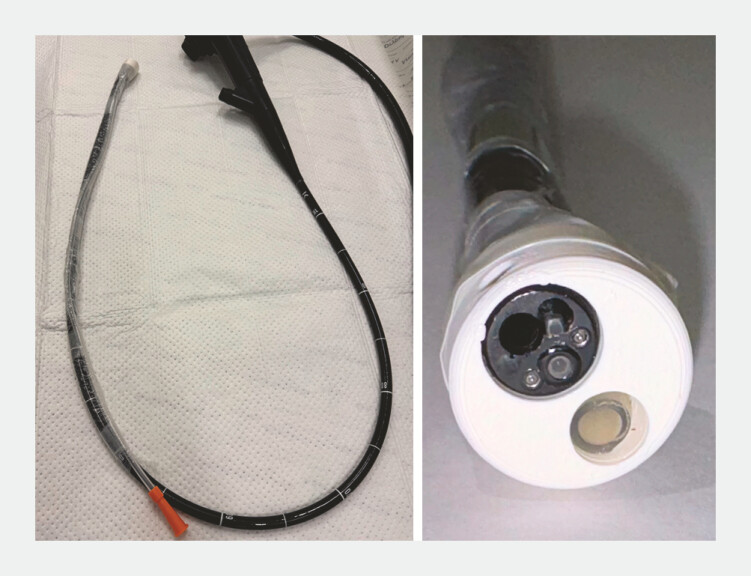
Standard gastroscope featuring an additional suction channel (ASC), secured with adhesive tape and a sleeve from the OVESCO AWC set. For enhanced visibility, a white first-generation cap with an enlarged size is depicted in the figure.

Standard endoscopes with working channel diameters of 2.8 mm, 3.7 mm, and 6.0 mm (Olympus Europa, Hamburg, Germany) were used. Due to wide availability of standard 2.8-mm gastroscopes we decided to use this model as our experimental foundation and added the ASC. Every endoscope was tested with and without the BioVac direct suction device (STERIS Endoscopy, Dublin, Ireland). The 2.8-mm gastroscope was equipped with a 16 Charrière (5.3-mm diameter) standard airway suction catheter, 60 cm in length (P.J. Dahlhausen & Co. GmbH, Colone, Germany), connected over a conventional fingertip with vacuum control (P.J. Dahlhausen & Co. GmbH, Colone, Germany) as the ASC. The catheter was mounted at the distal end of each endoscope using a custom-designed, 3D-printed polylactic acid (PLA) cap and secured with adhesive tape and a sleeve from the Ovesco AWC-Set (Ovesco Endoscopy, Tübingen, Germany) as shown in Fig. 1 . Negative pressure for the ASC was generated using a standard suction pump (MEDAP SEKRETSAUGER P 7030, ATMOS MedizinTechnik GmbH & Co. KG, Lenzkirch, Germany) set on maximal suction power, generation a negative pressure of 700 mm Hg. ASC suction pump was used together with standard suction pump of the endoscope tower (KV-5, Olympus Europa, Hamburg, Germany) also set on maximum suction power generating a negative pressure of 400 mm Hg via the endoscope working channel.

**Fig. 2:**
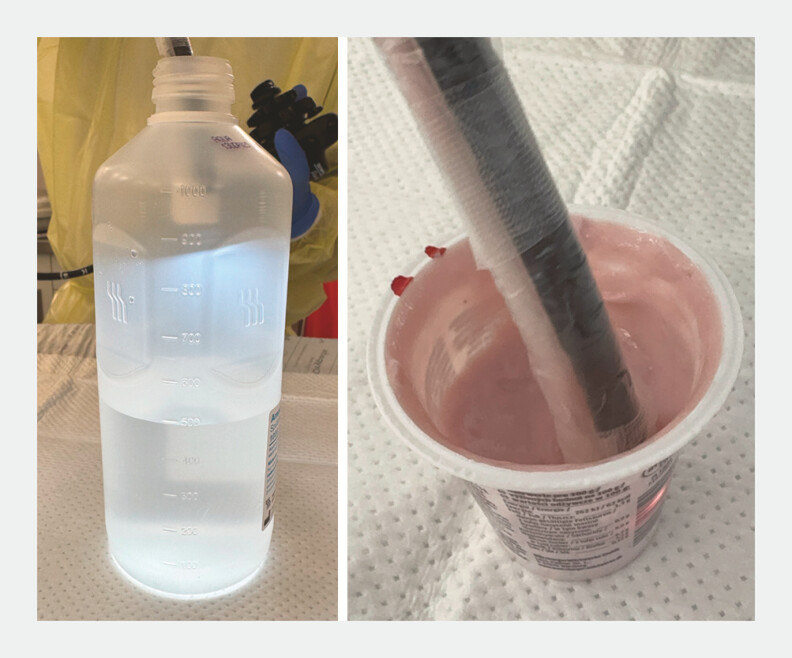
Endoscope handling while suction.

To evaluate suction capacity, 500 mL of water was initially aspirated out of a plastic bottle to determine maximum fluid extraction efficiency. Subsequently, the system’s performance was assessed using 125 g of room-temperature industrial fruit yogurt containing fruit pieces (Frankenland Top Fruchtjoghurt 3.5% Fett, Würzburger Milchwerke GmbH, Würzburg, Germany) as a medium with higher viscosity and solid particles. Fruit yogurt was sucked out of its plastic cup ( Fig. 2 ). Endoscope configuration and suction process are shown in Video 1 . Finally, 60 mL of clotted blood was aspirated from healthy departmental volunteers with previous blood donation experience. Therefore, blood was drawn under sterile conditions, placed in transparent plastic cups, and allowed to fully coagulate over a period of 3 hours. Due to missing data for power calculations, all experiments were carried out 10 times for each setup.

The time required for complete fluid removal during each aspiration trial was recorded. If the substrate could not be fully aspirated after 15 minutes, the trial was terminated. Endoscope occlusions were resolved by flushing the working channel or mechanical removal of clots or fruit fragments with a flexible working channel brush. Importantly, time measurements were not paused during occlusion clearance.

### Statistical analysis

Data analysis was performed using Prism 10 for macOS (Version 10.4.0; GraphPad Software, Boston, Massachusetts, United States). A Shapiro-Wilk test did not confirm normal distribution in all groups. Therefore, a non-parametric Kruskal–Wallis test was used for group comparisons with Dunn’s post hoc test to adjust for multiple comparisons. An adjusted *P* < 0.05 was considered statistically significant.

## Results

**Fig. 3:**
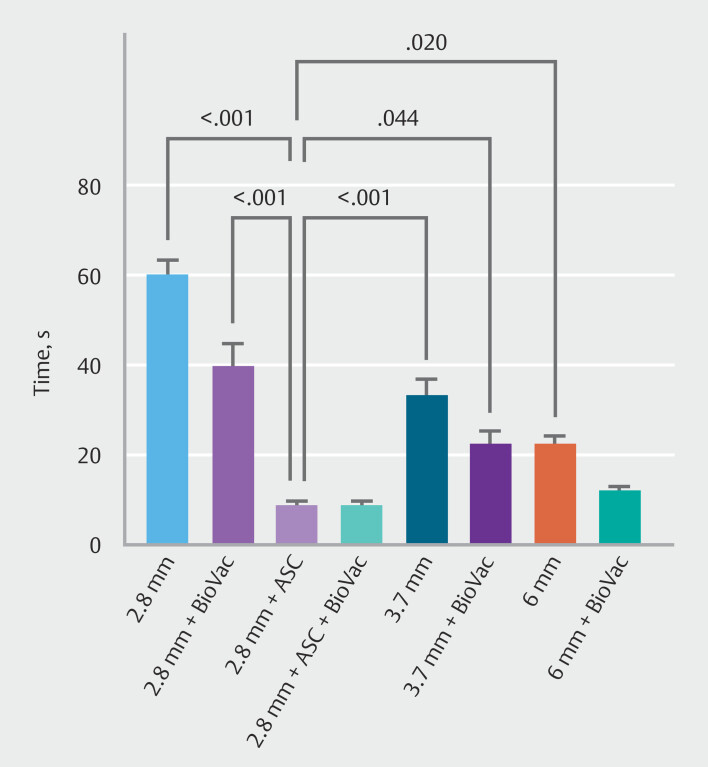
Time required to complete the suction of water using gastroscopes with varying working channel diameters, equipped with either the BioVac system or the BioVac system in conjunction with an additional suction channel (ASC). Only significant *P* values are shown in the figure.

The Kruskal–Wallis test showed significant differences between setups. Suction capacity for water using the 2.8-mm gastroscope with ASC was superior to all other configurations, except for the 6-mm gastroscope combined with the BioVac system. The addition of the BioVac system to the 2.8-mm gastroscope with ASC did not yield any further improvement in suction efficiency ( Fig. 3 ). In contrast, the combination of the BioVac system with the 6-mm gastroscope significantly enhanced water suction capacity compared to both the 2.8- and 3.7-mm standard gastroscopes. Furthermore, the 3.7-mm gastroscope equipped with the BioVac system demonstrated a significantly higher suction capacity compared with the 2.8-mm gastroscope without BioVac. In addition, the larger-channel endoscopes (3.7 mm and 6 mm) equipped with the BioVac showed a statistically significant higher suction capability compared with the 2.8-mm gastroscope. Finally, the 6-mm gastroscope was superior to the 2.8-mm gastroscope ( Table 1 ).

**Table:** **Table 1** Comparative analysis of gastroscopes with different working channel diameters, with and without the BioVac system, for the suction of water, *P* values of Dunn's multiple comparisons test.

Working channel of gastroscopes	*P* value
2.8 mm + ASC vs. 2.8 mm	< 0.001
2.8 mm + ASC vs. 2.8 mm + BioVac	< 0.001
2.8 mm + ASC vs. 2.8 mm + ASC + BioVac	> 0.999
2.8 mm + ASC vs. 3.7 mm	< 0.001
2.8 mm + ASC vs. 3.7 mm + BioVac	0.044
2.8 mm + ASC vs. 6 mm	0.02
2.8 mm + ASC vs. 6 mm + BioVac	> 0.999
2.8 mm vs. 2.8 mm + BioVac	> 0.999
2.8 mm vs. 3.7 mm	0,241
2.8 mm vs. 3.7 mm + BioVac	< 0.001
2.8 mm vs. 6 mm	< 0.001
2.8 mm vs. 6 mm + BioVac	< 0.001
2.8 mm + BioVac vs. 3.7 mm	> 0.999
2.8 mm + BioVac vs. 3.7 mm + BioVac	0.02
2.8 mm + BioVac vs. 6 mm	0.059
2.8 mm + BioVac vs. 6 mm + BioVac	< 0.001
3.7 mm vs. 3.7 mm + BioVac	0.376
3.7 mm vs. 6 mm	0.846
3.7 mm vs. 6 mm + BioVac	< 0.001
3.7 mm + BioVac vs. 6 mm	> 0.999
3.7 mm + BioVac vs. 6 mm + BioVac	> 0.999
6 mm vs. 6 mm + BioVac	0.553

**Fig. 4:**
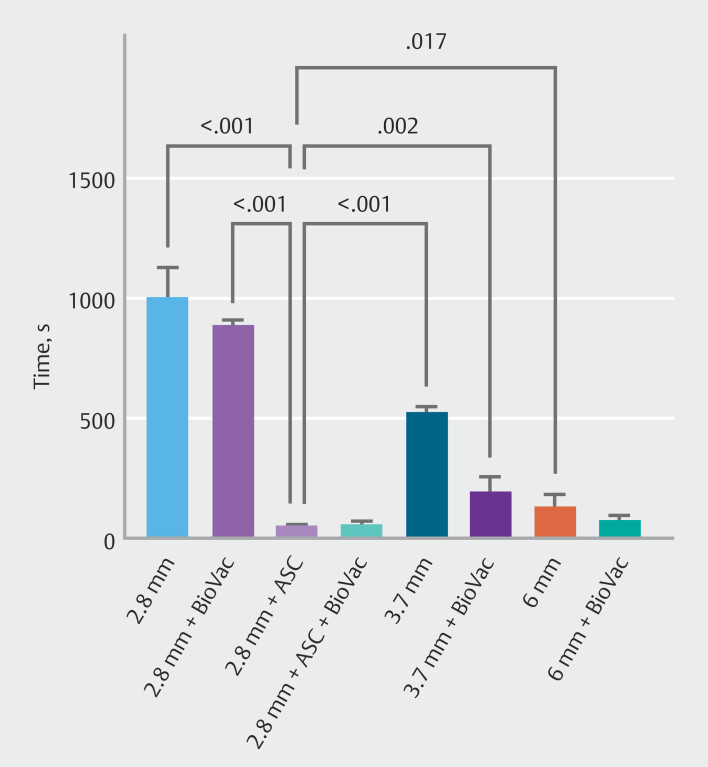
Time required to complete the suction of yogurt using gastroscopes with varying working channel diameters, equipped with either the BioVac system or the BioVac system in conjunction with an additional suction channel (ASC). Only significant *P* values are shown in the figure.

For fruit yogurt, which has a higher viscosity than water and contains fruit pieces, the 2.8-mm gastroscope with ASC again demonstrated superior suction performance compared with all other configurations, except for the 6-mm gastroscope with BioVac and the 2.8-mm gastroscope with ASC and BioVac ( Fig. 4 ). The 6-mm gastroscope with BioVac outperformed the 2.8-mm gastroscope (both with and without BioVac) and the 3.7-mm gastroscope without BioVac in terms of suction capacity. The 3.7-mm gastroscope with BioVac and the 6-mm gastroscope without BioVac were superior to the 2.8-mm gastroscope with and without BioVac ( Table 2 ). Suction times for yogurt were generally longer than for water, likely due to its higher viscosity.

**Table:** **Table 2** Comparative analysis of gastroscopes with different working channel diameters, with and without the BioVac system, for the suction of yogurt, *P* values of Dunn's multiple comparisons test.

Working channel of gastroscopes	*P* value
2.8 mm + ASC vs. 2.8 mm	< 0.001
2.8 mm + ASC vs. 2.8 mm + BioVac	< 0.001
2.8 mm + ASC vs. 2.8 mm + ASC + BioVac	0.794
2.8 mm + ASC vs. 3.7 mm	< 0.001
2.8 mm + ASC vs. 3.7 mm + BioVac	0.002
2.8 mm + ASC vs. 6 mm	0.017
2.8 mm + ASC vs. 6 mm + BioVac	0.247
2.8 mm vs. 2.8 mm + BioVac	> 0.999
2.8 mm vs. 3.7 mm	0.333
2.8 mm vs. 3.7 mm + BioVac	0.002
2.8 mm vs. 6 mm	< 0.001
2.8 mm vs. 6 mm + BioVac	< 0.001
2.8 mm + BioVac vs. 3.7 mm	> 0.999
2.8 mm + BioVac vs. 3.7 mm + BioVac	0.023
2.8 mm + BioVac vs. 6 mm	< 0.001
2.8 mm + BioVac vs. 6 mm + BioVac	<,001
3.7 mm vs. 3.7 mm + BioVac	> 0.999
3.7 mm vs. 6 mm	0.193
3.7 mm vs. 6 mm + BioVac	0.004
3.7 mm + BioVac vs. 6 mm	> 0.999
3.7 mm + BioVac vs. 6 mm + BioVac	0.570
6 mm vs. 6 mm + BioVac	> 0.999

**Fig. 5:**
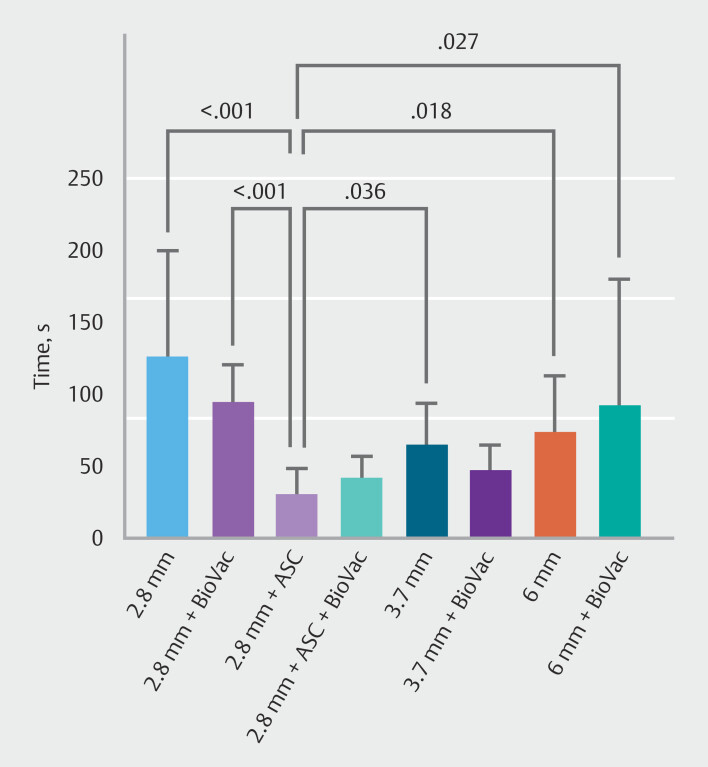
Time required to complete the suction of blood using gastroscopes with varying working channel diameters, equipped with either the BioVac system or the BioVac system in conjunction with an additional suction channel (ASC). Only significant *P* values are shown in the figure.

In the case of blood with coagulated clots, the 2.8-mm gastroscope with ASC demonstrated significantly superior suction efficiency compared with the 2.8-mm gastroscope (both with and without BioVac), the 3.7-mm and 6-mm gastroscopes without BioVac, and the 6-mm gastroscope with BioVac ( Fig. 5 ). No significant differences in suction times were observed among the remaining configurations for blood clot aspiration ( Table 3 ).

**Table:** **Table 3** Comparative analysis of gastroscopes with different working channel diameters, with and without the BioVac system, for the suction of blood, *P* values of Dunn's multiple comparisons test.

Working channel of gastroscopes	*P* value
2.8 mm + ASC vs. 2.8 mm	< 0.001
2.8 mm + ASC vs. 2.8 mm + BioVac	<0.001
2.8 mm + ASC vs. 2.8 mm + ASC + BioVac	0.512
2.8 mm + ASC vs. 3.7 mm	0.036
2.8 mm + ASC vs. 3.7 mm + BioVac	0.250
2.8 mm + ASC vs. 6 mm	0.018
2.8 mm + ASC vs. 6 mm + BioVac	0.027
2.8 mm vs. 2.8 mm + BioVac	> 0.999
2.8 mm vs. 3.7 mm	> 0.999
2.8 mm vs. 3.7 mm + BioVac	0.190
2.8 mm vs. 6 mm	> 0.999
2.8 mm vs. 6 mm + BioVac	> 0.999
2.8 mm + BioVac vs. 3.7 mm	> 0.999
2.8 mm + BioVac vs. 3.7 mm + BioVac	0.190
2.8 mm + BioVac vs. 6 mm	> 0.999
2.8 mm + BioVac vs. 6 mm + BioVac	> 0.999
3.7 mm vs. 3.7 mm + BioVac	> 0.999
3.7 mm vs. 6 mm	> 0.999
3.7 mm vs. 6 mm + BioVac	> 0.999
3.7 mm + BioVac vs. 6 mm	> 0.999
3.7 mm + BioVac vs. 6 mm + BioVac	> 0.999
6 mm vs. 6 mm + BioVac	> 0.999

## Discussion

A clear visual field is critical during emergency endoscopy to identify and treat bleeding sources effectively. Our findings offer valuable insights into the suction capabilities of various gastroscope configurations under different conditions, including water, fruit yogurt, and blood with clots. The findings indicate that the standard 2.8-mm gastroscope with an ASC demonstrates superior suction performance compared with special gastroscopes with larger working channels.

Special gastroscopes featuring larger working channels were developed and commercially introduced over 30 years ago 3 4 . However, their limited availability due to high costs and restricted clinical applications has hindered widespread adoption.

Optimization of suction power using a separate suction hose and pump system, such as the BioVac system, was described in the late 20th century 9 and is commercially available today. However, to best of our knowledge, we found no independent evaluations of the BioVac system for upper gastrointestinal bleeding. The only available data derive from a small manufacturer-led study, which was not published in a peer-reviewed journal 22 and reported a higher suction capacity compared with standard gastroscopes. However, our study was unable to reproduce these findings. Depending on the aspirated medium, we observed a slightly higher suction capacity in most cases, but the differences did not reach statistical significance. One study demonstrated a significant advantage of suction through the biopsy port over conventional suction via the standard connector at the endoscope interface with the video processor and light source 8 . That study, which also used yogurt as a high-viscosity test medium, did not provide information about presence of fruit pieces.

A more recent study evaluated a modified overtube combined with a nasal endoscope for removal of gastrointestinal residue in both in vitro and in vivo settings 17 . The authors reported superior suction capacity with the overtube compared with a standard gastroscope. Like our results, the modified overtube effectively cleared test substances with higher viscosity and larger solid components. However, unlike our ASC system used with a standard gastroscope, the nasal endoscope’s smaller working channel severely limited its capacity for therapeutic interventions, such as endoscopic hemostasis. With the ASC in place, the working channel remains free, enabling introduction of devices such as clips or injection needles at the same time. However over-the-scope clips could not be deployed with either system.

Suction capacity generally depends on fluid viscosity, vacuum power of the pump, and length of the suction hose, as described by the Hagen-Poiseuille law. Gastrointestinal residue and blood clots frequently cause occlusions in the endoscopic working channel or suction hose. In addition, we observed fingertip occlusion due to the small diameter of the standard fingertip control, which remains a problem that requires further technical solutions. In addition, endoscope obstructions caused by fruit pieces were more frequently observed in endoscopes with narrower working channels.

Our study has certain limitations. It was an in vitro feasibility investigation and the selected blood volume was smaller than typically encountered in clinical practice. Nonetheless, it was sufficient to demonstrate differences between experimental groups. In addition, our 3D-printed cap had a diameter of approximately 18 mm, although there is potential for further size reductions in future revisions. The standard airway suction catheters used in the study were limited in length and would need to be replaced with longer models for effective use in duodenal coagulum removal. We also did not investigate the limitation in field of view by our PLA-cap and the influence on endoscope handling. This has to be investigated in the future in an ex vivo model. Theoretically the ASC can be attached to larger endoscopes but resulting in a larger diameter of the whole construct. Clinical use could be limited by the larger diameter.

## Conclusions

In summary, our findings suggest that adding an ASC to a 2.8-mm gastroscope significantly enhances suction capacity in scenarios involving water and blood clots, outperforming even larger endoscopes, particularly for materials containing solid components such as clots. While a 3D-printed cap is helpful, it is not essential—the suction catheter can be directly taped onto the endoscope as long as the catheter tip remains visible and navigable in front of the endoscope tip. We propose that the ASC represents a simple, cost-effective, and versatile solution, because it can be attached to any standard endoscope. In settings where larger-channel endoscopes are not available due to limited accessibility, the ASC, therefore, may enable hospitals to benefit from its advantages, thereby enhancing suction performance of standard endoscopes without the need to acquire additional equipment. Further ex vivo studies are necessary to validate these findings and evaluate their clinical applicability in more realistic settings.
